# Methodological reflections on artistically illustrating ethnographic text from a study of sport pedagogy in youth detention: Ethics, affect, and description

**DOI:** 10.3389/fspor.2022.1021915

**Published:** 2022-11-10

**Authors:** Daniel Roe, Åsa Bäckström

**Affiliations:** Department of Movement, Culture and Society, Swedish School of Sport and Health Sciences, Stockholm, Sweden

**Keywords:** illustration, ethnography, sport pedagogy, affect, ethics, thick description, youth detention, total institutions

## Abstract

While there is a burgeoning body of literature on visual methods in ethnography, including drawing and illustration as method for collecting and exploring data, little has been written about how artistic illustrations can be used as a representational method for finished ethnographic texts. Based on an illustrated ethnography–a PhD thesis on sport pedagogy in youth detention–this paper explores what artistic illustrations can do for representations of ethnographic texts. An important starting point of the paper is that artistic illustrations are purpose-full–they can be used strategically to highlight some details over others, thus making it possible for researchers to selectively accomplish several aims when it comes to representation. Particularly, we focus on ethical, affective, and descriptive purposes for using artistic illustrations when publishing research on sport in total institutions. We present selected illustrations from the thesis together with analytical and procedural commentary to shed light on some strategic thinking behind the production of the illustrations. Finally, we conclude with some reflections on the methodology and discuss some further considerations for illustrating ethnographic texts in terms of benefits, risks, and possibilities.

## Introduction

Ethnography is both a method of in-depth study and a representational form or genre; a process and a product (1, 2). In other words, ethnography is an approach to accessing, examining, and documenting social life at the same time as it is the production of “thick” textual descriptions and analysis (1, 3, 4). These two aspects can be related to significant rationales for ethnography of prisons or carceral settings, including youth detention: to access and investigate these closed social systems, and to contribute subtle variations, descriptive, and humanizing accounts of life in such places. Because youth secure care settings are generally closed to the public eye, ethnography can play an important role in accessing and investigating the practices of these total institutions, offering nuanced and critical insights that can inform efforts to safeguard the rights and wellbeing of the young people confined within (5). Ethnographic accounts of life in youth detention can enrich our knowledge for how youth detention homes operate and can contribute sophisticated insights, especially pertaining to a group of youth who are dehumanized, stigmatized, and criminalized both in broader discourse as well as within institutional settings (5, 6). In this paper, we will elaborate how artistic illustrations can further and accentuate such knowledge contributions.

Although photography and film has been used in ethnographic research for quite some time [e.g., (7)] and ethnographic film has gained recognition and developed into a genre in its own right [e.g., (8)] ethnography is still largely a “textual enterprise” (1), and published versions of ethnographic research consists predominantly of typed words. In recent years, however, we have seen an increase and diversification in the use of visual methodologies in ethnographic research (9–11), including research in sport [c.f. (12, 13)] and sport pedagogy (c.f. (14)). Scholars have pushed the boundaries on how to incorporate various visual methods when collecting, analyzing or exploring data [e.g., (13, 15–17)]. There have been various reasons to do so, including to make small children's social relations and practices visible (15), as a technique to enhance reflective practices (13), as a way to probe reactions among research participants and the researcher (17), or as a way to disseminate research findings in an appealing and simple way in order to empower and educate readers (16).

When it comes to dissemination, one particular form has gained widespread and “trending” popularity – graphics (18). The rationale for the increasing acceptance is its “capacity to cut across a wide age-range, literary levels and socio-economic and socio-cultural diversity” [(18), p. 293]. According to Boudreault-Fournier (19), graphic novels have the potential to “explore new avenues of research creation and dissemination” a lesson learned from teaching anthropology inspired by McCloud (20) and others. The qualities of comics are defiantly valuable. Yet graphics may for example connote ease and humor, somethings which researchers may not always wish to communicate. In this paper, we describe and reflect upon methodological considerations when illustrating a PhD thesis, an ethnographic study of sport pedagogy in youth detention (21). Doing so, we add to the discussion on what visual and artistic aspects can contribute to the dissemination of social research in ways which at times must be devoid of the ease and humor of comics. The questions we address are: what were the main considerations undertaken when illustrating this ethnography of sport in youth detention and, what are the illustrations *doing* for this ethnography, and perhaps other ethnographies? Indeed, this is certainly not the first time an ethnographic text is illustrated. Yet there is dearth of description on the considerations and processes of doing so. How does one go about illustrating ethnography? What considerations and decisions are undertaken in that process? Linghede et al. (22) write:

It is as if the writing of stories is some kind of mysterious abracadabra activity and not a deliberate and theoretically informed creative process. And maybe the construction of stories has not been given enough attention. Maybe there has been too much of a focus— though utterly important—on the potential of, the philosophical foundations of, and the criteria for judging narrative research (p. 84).

In a similar way, this paper is an attempt to bring the reader and curious researchers “under the hood” of what went on in the “abracadabra” process of producing illustrations for ethnography.

To do so and to answer the above questions, we bring together perspectives from visual ethnography [e.g., (9)], representation in ethnography [e.g., (23)], creative story-telling and narrative methodologies [e.g., (22)], and perspectives on illustrating scholarly works [e.g., (16, 24)]. These perspectives are reflective of the authors' experiences and contributions to this methods paper. Daniel (Dan) is the primary researcher; his research/findings informed the illustrations. Åsa was an informal mentor to Dan during his PhD project, her experiences with ethnography and visual methods help inspire and inform some of the key considerations described below. The artist, Jenny Soep, completed the illustrations, her insight on the artistic side of illustrating the ethnography are reflected throughout the text in her images and as a secondary source.

This paper is organized as follows. Initially, we will discuss selected examples of using visual material in disseminating qualitative research. Next, we provide an overview of the thesis itself, its method, analytical considerations, and main themes. The overview is needed to contextualize further discussion on the illustrations. Thereafter, we describe the main methodological considerations when undertaking to illustrate the thesis: *ethics, affect, representation, meaningfulness, logistics/resources*, and *style/aesthetics*. This is followed by a presentation of the three key themes for this ethnography which shows selected images themselves, accompanied with analytical/theoretical and more detailed accounts of the procedure of creating the illustrations. The three themes: ethical, affective, and descriptive, show how purposeful use of illustrations may be combined with research findings to communicate various results in a conscious, creative, and compassionate manner. Lastly, we discuss the approach to illustrating ethnography, touching upon issues of voice, the creative process, representation, and credibility. The discussion also poses considerations for others interested in illustration as a visual method in ethnography.

## Illustrating research—Current issues

The benefits of visualizing data are manifold. Grodoski [(25), p. 39] argues that both data and theories may be summarized in an “aesthetic and understandable manner” and that “visualized data are a powerful means […] for a variety of disciplines.” Illustrating ethnography or other types of research may be done in different ways depending on intentional communication, but research epistemology always plays a crucial part in decisions and processes in all stages of the research. Van Maanen's (23) *realist* tale describes a way to, as objectively and realistically as possible, picture the researched phenomenon. His *impressionist* tale rather describes a way to invite the reader to personal accounts allowing for an understanding of the “feels” of the data. One field in which the increasing popularity of graphic arts as a way of disseminating research has been considered, used and discussed is public health [e.g., (16, 26, 27)]. For instance, Febres-Cordero et al. [(16), p. 42S], argue from a realist standpoint that the dissemination of research findings in a “culturally appropriate graphic and narrative language” will help decrease mortality from opioid epidemic. The realist epistemology, as well as the polarity between realism and impressionism may however be complicated when using artistic illustrations. Herbst [(24), p. 80], describes her fourpage comic “Life of Lola” as “a distilled 2 years of fieldwork, six field journals, two diaries 77,218 word manuscript and the daily lived experiences of 31 children and young people diagnosed with Medium-Chained Acyl CoA Dehydrogenase Deficiency (MCADD).” In other words, the graphic draws from rigorous scientific data in the real world with real people, using real hardcore medical terms, but it also communicates research findings in an accessible “feely” way to academics and non-academics alike.

Advocating for the use of aesthetic based methods and artwork to communicate research findings, Glass (28) shows how vulnerability came across in the art exhibition following her first ethnography. This aspect was important for involvement with the audience and opened up for a willingness to engage and share their thoughts and feelings. Reciprocity, as defined by Van Maanen [(29), p. 113–114] as “knowledge [which] is not available to our linguistic competency [but] put into words by another person” became crucial when viewers contributed with new thoughts to the researcher. Art-based methods have the potential to “stimulate reflexivity,” according to Archibald and Gerber [(30), p. 972], which in turn “is needed across research traditions to cultivate awareness, both to overcome conceptual challenges and to avoid “automata” – a tendency to take on practices and terminologies because they are established rather than conceptually coherent given the state of empirical and theoretical understandings within a discipline.”

Herbst [(24), p. 81] states that “the comic's main contribution is in terms of representation.” It is a way to illustrate the everyday and make sense of what makes us human. Not least are affective considerations important to include, as they are key to authentic and nuanced analyses. Here, participant's voices may be heard, especially those voices who risk being muted. Representation becomes specifically important to consider when working with an external illustrator. To Febres-Cordero et al. (16), it was imperative to disseminate the results both in ways that were authentic, ethical, inclusive and engaging. To them, the process of working with a local graphic artist, for instance included adjusting skin-color in order for the research participant to feel included.

## The study in question—Ethnography of sport in youth detention

This article draws on Dan's doctoral thesis (21), an ethnographic study of sport at two all-male youth detention homes in Sweden (ages 16–21). The specific aim of the thesis was to explore pedagogies of sport in youth detention. An overarching research question was: How is sport arranged, delivered, and experienced in youth detention? These descriptive questions endeavored to illuminate what roles sport pedagogies play within youth detention, with a particular interest in how sport can affect the life situations of detained youth. The research involved two sub-studies, totaling over 500 h in the field. Fieldwork was immersive, and primarily involved participatory observations of life at both institutions, with a particular focus on sports and physical activities. Sports and physical activities took place in school, during recreational time, and with outsiders (namely a local sports club where some of the boys played football). During the studies, Dan also conducted semi-structured interviews with both youth and staff, where 39 students and 32 staff members were interviewed.

The thesis was published as a composition thesis, based on three independent but interrelated articles (31–33). Data analysis in the research has some important commonalities: a similar process of coding, writing and re-writing; close description; grounding in student experiences; and seeking to craft vivid, human connections between the reader(s) and the worlds of these young men. In all the analytical work, the coding sought to highlight key experiences (particularly from the students' perspective) and ways of delivering sport (including how the structure and delivery was rationalized). This enabled Dan to collate student experiences and look for patterns or relations with ways of delivering sport. Trying to interpret how these two dimensions were possibly related formed the basis and guided the direction for all the analyses. The interplay between pedagogies and experience was likewise a key consideration when illustrating the thesis (i.e., that the images should reflect both key experiences and the pedagogies of youth detention and sport within).

The principal contribution of the thesis is to show *how* different sport pedagogies function to educate or support and, conversely, contain or punish placed youth – showing how desirable benefits of sport in youth detention are contingent upon pedagogical practice. The thesis describes experiences of doing sport in youth detention, particularly from the young people's perspective, and connects their experiences to the pedagogies of institutions and sport within them. Some of the key findings from the thesis are that *s*port is experienced in different, often contradictory ways by the youth, for example as boring but sometimes exciting and fun, punitive but also empowering or liberating, and as simultaneously inclusionary and exclusionary. Sport is also withheld in terms of access and quality to correct or control youth and thus be likened to a pedagogy of corrections or punishment. At the same time, sport is a pedagogy where youth experienced learning new perspectives and having increased opportunity in life through their participation in sport. It can be thought of as a canvas, or platform, for a critical pedagogy.

Dan contacted several artists about illustrating the compilation text for the PhD thesis. Even with a limited budget, four artists expressed interest in the project. Dan browsed the portfolios of each artist and ultimately chose Jenny Soep for a style that he desired for the envisaged images: lines that were clear but not sterile, and a balance of warmth, youthfulness, and professionalism. When Dan described the research, Jenny shared that she could relate *via* her experiences training martial arts in an impoverished part of the city. To commission the illustrations, Jenny asked Dan to send her a PowerPoint that described the envisaged drawings together with drawings from her own portfolio which could help her understand the desired style or feel of each image. She also requested that Dan send some of his research so she could get a feel for the people and places she would illustrate.

## Illustrating the ethnography—Some key considerations

In this section, we categorize the main considerations behind illustrating the thesis in terms of *ethics, affect, description, meaningfulness, logistics/resources*, and *style/aesthetics*. Before we go into further detail about these considerations, it is important to note that these categories should be thought of as intensely interrelated and are not neatly separated. Indeed, they can hardly be ranked or ordered (and the order of presentation below should not be interpreted so). Rather, this interrelatedness is indicative of the messiness and complexity of doing and writing ethnography (1, 23). The concept of *access*, for example, cuts across many of these categories. First, the illustrations can be seen as visualizing the textual descriptions of the data and analysis, i.e., to access the thick description in the text through visualization. Secondly, they enable unfamiliar readers to access or to re-access the context through feeling and emotion. Thirdly, as we will discuss below, this kind of affective immersion is a point of ethics in researching incarcerated youth, in that researchers should not detach themselves from the emotional experiences of researching young people in vulnerable life situations [see (5)].

**Figure 1:**
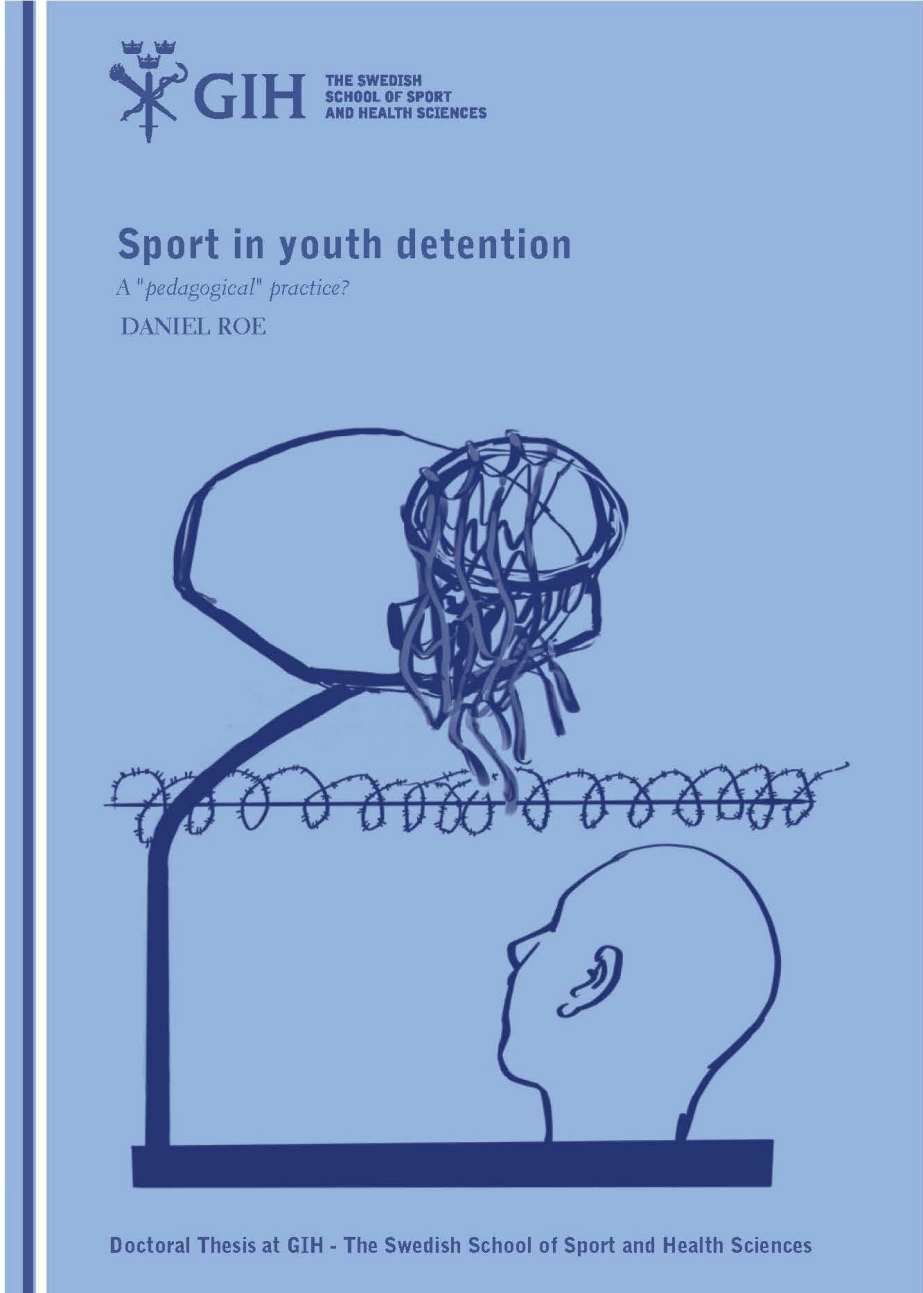
Front cover of the thesis. Illustrated by Jenny Soep.

At the beginning of each chapter in the PhD thesis (21), illustrations inspired by scenes from the field work were featured. All in all, there are eight artistic illustrations including the one on the cover. The main considerations with every illustration and in particular the cover were simplicity, relatability, and questioning. For the cover illustration, see Figure 1, the aspiration was to convey in a simple, relatable way that the thesis is about sports in a prison-like setting. For example, Dan has been to many prisons and youth detention homes, both in Sweden and the United States, and a basketball hoop with tattered net (typically in a common recreational space) is something one could find in practically any youth detention home. To clearly convey the prison-like setting, the basketball illustration also shows barbed-wire in the background. While each image in the paper is based on the author's field experience, the author also intentionally selected more abstract drawings to be relatable in other international contexts.

The main intention was to prompt the reader to ask questions about sport in youth detention, for example: What is it like? How is sport delivered in this setting? Are they being used to “rehabilitate” detained youth? Also, sports are not being taken advantage of or delivered to their fullest potential. The angle of the image, with the figure looking up to the hoop, is intended to convey a sort of questioning – a quizzical angle. The title of the thesis is, itself phrased as a question. It questions the ways sports are delivered and experienced in youth detention. It also asks whether sports are creating opportunities for detained youth, and if they are underutilized as a “pedagogical” practice. Here, the purposes of the illustration are to reflect the theoretical and philosophical underpinnings of the thesis' argument, while also provoking readers to ask these questions themselves. Open the cover, read on, and this ethnography will shed light on those questions.

Excluding the cover image, the illustrations reflected the three dominant pedagogies of sport described in the thesis – withholding sport (sport as corrections and punishment), busying with sport (sport as containment and “filling the time”), and sport as developmental community (sport as a platform for learning and development). As shown below, the illustrations also capture marginalized masculinities in relation to sport. Most of the images have been generated at least in part from photographs that were collected during the field work. When the thesis was first published, there were no substantial descriptions or reflections on the methodological considerations with the illustrations, other than to briefly describe them as supplementing the text.

One consideration was that the images should “speak” for themselves. This “speaking,” however, was informed by dialogue between author and artist during the process of creating the illustrations. Also, the process of working on the illustrations together with an artist spiraled further reflection and analytical understanding as both Glass (28) and Archibald and Gerber (30) point out. Although the illustrations were left without captions in the thesis, so as not to “rob” the readers of their own interpretations, we would like to acknowledge the ambiguity and multiple possible meanings available^1^. This was intended, especially for practitioners or stakeholders, so that the images sparked personal pedagogic reflections (34).

The selection, production, and presentation of the images was purposeful (9); the illustrations were intended to help convey or visualize important themes, findings, and context from the project, but also much more. For example, because the research settings – secure youth detention homes – were closed and obscured from public view, the illustrations aimed to invite readers to access and reflect upon the worlds of sport in total institutions (35).

In the following sections, we will describe some of the three key analytical considerations behind the images, with the caveat that some, indeed many of the considerations undertaken in illustrating the thesis will be left unsaid. The analytical commentary will be completed with some procedural descriptions.

### Ethical purposes

While photography and film have the potential to capture “real” events as they play out, including bodily movement, posture, and facial expressions, thus allowing for credible data (in terms of validity and reliability as positivists would put it), the possibility to identify individuals makes data in the form of photography and film problematic from an ethical point of view. Incarcerated young men in this setting are particularly vulnerable. The use of illustrations, as opposed to photographs, has the benefit of highlighting particular details (i.e., to capture certain themes or feelings) while also leaving out details (e.g., to ensure confidentiality). One of the first considerations for using illustration in the thesis was as a way to include visual artifacts from the data collection. During the field work, some visual material (mainly photographs) was collected, some of which Dan took, and others provided by the institution. However, to use them would be problematic due to confidentiality concerns. Photographic realism might reveal too many details about either the participants or the sites of the research. Using illustrations as opposed to photographs allowed Dan to control the details and information on the images, and to help ensure the confidentiality of the youth and the research sites. One possibility could have been to use censored photographs, i.e., to blur or put bars on the faces. This is a common way to picture “young criminals” placed side by side with crime scenes. Such an approach risked further criminalizing the participants. The latent ethical purpose not to do this rests on the decision to not dehumanize the boys. There are subtle and explicit ethical risks in researching incarcerated youth wherein the researcher, albeit unknowingly or unwillingly, might further dehumanize or stigmatize the participants. In this research, the ambition was to show the young people as they were experienced during research, as vivid human beings, as *students*. For this reason, the youth participants are referred to mainly as “the boys,” “students,” or “youth,” and labels such as “criminal youth,” “young offenders” or “inmates” have consequently been avoided.

**Figure 2:**
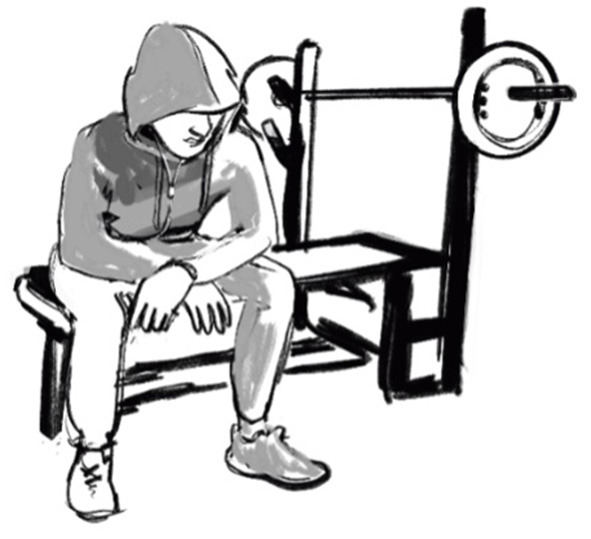
Alone at the gym. Illustrated by Jenny Soep.

As an example of the ethical purposes, we have chosen to discuss an illustration of the gym practice (Figure 2). In this illustration, Dan wanted to show the isolation or marginalization experienced by the boys, but also the inability of sport pedagogies in the gym to meaningfully engage the boys and address their marginalization. These moments of vulnerability and exclusion which we observe and feel in the field [c.f. (5), p. 842-843] are hard to capture photographically. Furthermore, the boys in the study shielded their vulnerabilities from others and were hesitant to show them to Dan (a relatively young male researcher).

The posture is a common feature and could be attributed to any one of the boys included in the study, as is the hood which covers much of the head and the face. These features allowed for both recognition and confidentiality. Besides the gym context placing the activity and person in this material surrounding, the posture, with spread legs and heavy shoulders, has a masculine connotation. One of the three papers in the thesis was about sport and masculinity in youth detention, and with this image the idea was to convey “marginalized masculinity.” This paper described how the gym activities of many of the boys were connected the marginalized masculinities of young men and boys placed in detention.

Theoretically, we (Roe and Larsson, unpublished manuscript) approached masculinity as being relationally and socially constructed, hence using the plural use of masculinit*ies* (36). Furthermore, we viewed the students' masculinities in terms of marginalized masculinities, i.e., that the boys' ways of perceiving gender mirrored marginalized positions [see e.g., (37)]. For many young men and boys, incarceration perpetuates experiences of being seen as threats [see (38, 39)], further marginalizing them through denying or otherwise failing to provide means for fulfilling (narrow) expectations of independent adult manhood (37). With this picture, we wanted to emphasize the loneliness, exclusion, and disengagement associated with marginalized masculinities. To begin with, the gym practice at one of the institutions (Summerholm) was typically a lonely practice:

On most occasions, one staff member accompanied one or two students to use the weight room. Such sessions were often low intensity, and without much student-staff interaction; staff members mostly did not work out with the students.

Furthermore, in the thesis this pattern of gym practice was connected to a pedagogy of withholding sport, and a discourse of “how good should they have it?” that disengaged practitioners from high quality sport pedagogical meetings.

When considering what and how to illustrate “masculinity” in youth detention, there was ample data to choose from. The topic itself – sports in all-male youth detention homes – lends itself to the entire ethnography being saturated with masculinity (and all the illustrations could easily be interpreted through a masculinities lens). That said, one scene in particular from Article 3 stands out, where one student used the gym to express hypermasculine norms connected to criminality, violence, and the use of anabolic steroids:

In between sets, [Jason] adds more weight and flexes, watching himself in the mirrors, and practices looking hard and aggressive. He mimics throwing punches with a clenched jaw, and makes various intimidating poses and gestures… He continues the hard looks into the mirror, sometimes taking his shirt off.

Jason tells stories about violence he has witnessed, a fight, a shooting, acting out the events and laughing. Jason boasts about taking steroids to get bigger, explaining how the pills allow one to lift more and lift longer.

The interpretation was that such displays are concerning not least because they appear to put these boys in situations where they might harm themselves or others, but also as potentially alienating them in future social interactions and circumstances. Furthermore, visualizing Jason's performance risked overemphasizing the students as threats, and underemphasizing the social isolation/exclusion of doing marginalized masculinities. As Rios (37) concludes, doing hypermasculinity can feel empowering in the moment, but has exclusionary consequences later in life. The chosen illustration goes beyond this type of stereotyped hypermasculinity and allows for an ethical handling of the research participants and for a different masculinity to be interpreted.

### Affective purposes

Affective immersion in field work often blurs together with research ethics and representation [c.f. (5)]. One of the inspirations for doing the illustrations was a creative-narrative approach to researching sport. Similar to the narrative methodology of Linghede et al. (22), we believe illustrations can “…embody knowledge in a way that hopefully will reverberate with the reader not only as information, but also as emotions and desire” (p. 83). In describing this world of doing sport in youth detention, Dan hoped that readers can connect with and see students as he did, not as distant others, but as vivid human beings. Although he has tried to provide an accurate and unbiased account of experiences from the field, how he sees and writes about students has without a doubt been influenced by his affective immersion in the field (5). While it is important to acknowledge potential bias and subjectivity, affective immersion is also a powerful source of pedagogical reflection. Van Manen (29) writes: “The aim of phenomenology is to transform lived experience into a textual expression of its essence - in such a way that the effect of the text is at once a reflexive reliving and a reflective appropriation of something meaningful: a notion by which the reader is powerfully animated in his or her own lived experience” (p. 36).

Some of these scenes from the field have been so starkly imprinted on Dan that he quite simply could not ignore the desire to in some way try to reproduce them. These memories and feelings could be considered “insight cultivators” (29) in the analysis and writing of the thesis. Furthermore, the illustrations are intended to affect sport pedagogy in youth detention – that is, they are meant to stimulate thinking about how sport is structured, delivered, and experienced in youth detention. Similar to Herbst [(24), p. 81], who wanted the reader to “climb inside the body and the mind of these children, to feel what they are feeling,” Dan wanted his passion and experience for the topic, as well as genuine interest in this world, to bleed through the texts. The same can be said when creating the illustrations.

**Figure 3:**
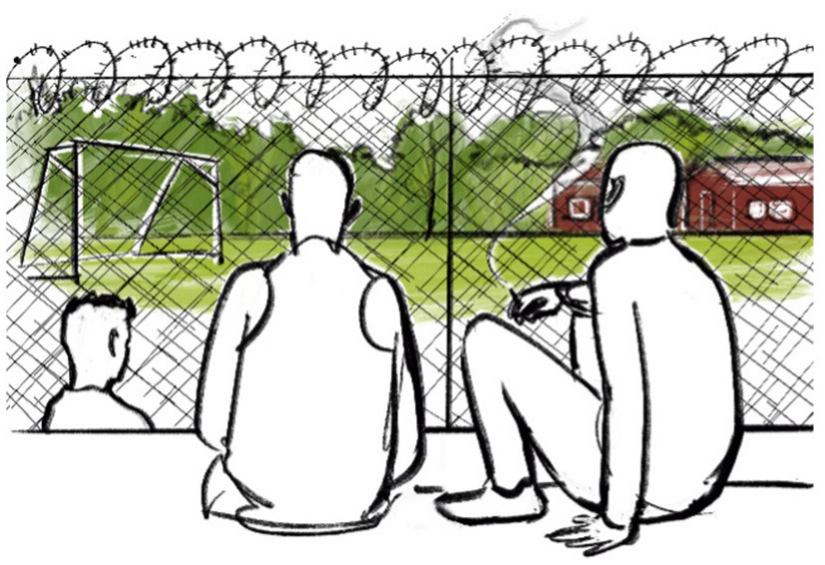
Smoke break. Illustrated by Jenny Soep.

As an example of the affective purposes, we have chosen to discuss the illustration of a smoke break (Figure 3).

This illustration relates to how pedagogies of youth detention are limiting youths' access to meaningful experiences of sport. As with Figure 2, this image helps to visualize the large amounts of time spent in youth detention NOT doing sports or yearning to do sports but not being able to. In the experience of Dan, practically all youth detention homes have serviceable sports facilities, fields, or outdoor spaces that tend to be severely underutilized [see also (40, 41)]. But perhaps the most important thing about this image is how it captures the affective domain: the desires from many of the boys to do sports, while being withheld from doing so. Arguably, it is the desires of the boys to play sports, have fun, and improve themselves (as people but also as athletes) that is the most important untapped resource – opportunity – for improving sport pedagogy in youth detention. The details of the artwork are conveying that we are looking at sport – and sport pedagogy – as something that *could be*. The artist's use of color in this image affects this kind of reflection and also the body language of the boys, especially the boy on the left. His posture communicates how relentless boredom and social control can numb young people's interests and desires.

In the thesis, desires and abilities of the boys to do sport was met through institutional practices that failed to act upon such desires as pedagogical opportunities [see, e.g., (42)]. This image was placed at the beginning of the discussion in part to summarize how dominant pedagogies of withholding sport and busying the youth hardly can be considered “pedagogical” practices, in that they lacked clear beliefs and aims regarding educating, developing, or otherwise creating life opportunities for the boys beyond social control at the institutions. The other part of this choice was to affect thinking about the pedagogical opportunities of sport in youth detention. Dan wanted the readers to desire, as he did and as the boys did, to get out from behind that fence and do something! To go run around on that football field, play, have fun, to be “kids.” This desire acknowledges that sport is there – it is waiting to be developed as an effective pedagogical practice in youth detention. Furthermore, Dan hopes that the work inspired pedagogical thought as to how this could be done: How can we dismantle the barriers to meaningful sport in youth detention, i.e., the fences between the boys and the football pitch? What would sport practices with more clear pedagogical purposes look like?

### Descriptive purposes

Adding the illustrations let Dan strike a balance between the thick description of the context and a selective focus on salient themes from the ethnography. Arguably, one of the main concerns with ethnography is thick description (4). It is precisely the close descriptions from the field in other ethnographic monographies [e.g., ((43), p. 47)] that captivated and intellectually engaged Dan in the life situations of the young men. He sought to craft thick descriptions (4) in all three articles, through the use of vignettes and/or frequent interview citations, which bring the reader closer to lived experience and the character of the pedagogical practices. But at the end of the process of writing the composite thesis (a collection of articles), Dan had the sinking feeling that the kind of dense description employed in the monograph genre was not accessible to a broad readership. “Language has its limitations,” as Herbst [(24), p. 81] puts it.

Another consideration related to representation was how the illustrations might add – or perhaps even risk – the trustworthiness (validity) and credibility of the text. Cunliffe (44) writes that “[e]thnographic validity is not determined in the same way as scientific validity but is instead based on the credibility of the text: is the text authentic, conveying a sense of the ethnographer being there and grasping the intricacies of life in that setting?” At the risk of being overly self-congratulatory, the illustrations were praised by Dan's thesis opponent, who asked about them as her final question of the defense. “Are illustrations typically featured in Swedish doctoral theses?” The opponent appreciated this touch and commented that they reminded her own experiences in the field researching sport in prisons. In other words, the use of illustrations seemed to work for her and other readers and was thereby validated in academic circles. Similar feedback was given from staff in the youth homes who commented that they appreciated recognizing the situations depicted in the images and the style of the illustrator. One staff appraised the style as “warm” and “not too serious.” Some of the images were based on photographs that Dan used when presenting and discussing his research results on previous occasions at one of the institutions.

**Figure 4:**
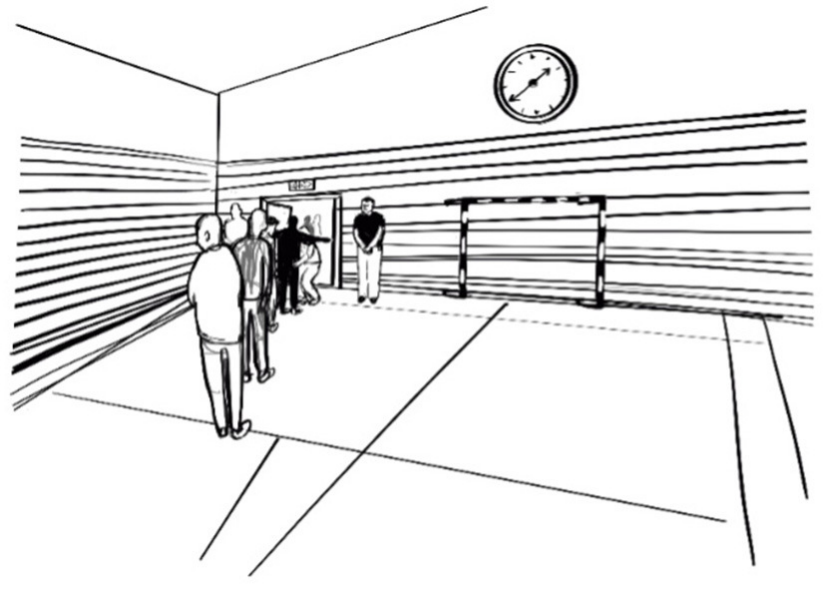
Leaving the gym. Illustrated by Jenny Soep.

As an example of the descriptive purposes, we will discuss the illustration of what leaving the gym looked like (Figure 4). This image was strategically placed at beginning of the thesis, corresponding to an opening vignette about a basketball training at a youth detention home in the United States. The vignette intends to bring the reader, immediately and vividly, into the context of sport in youth detention. In the opening, a similar scene is mentioned, where students line up to leave the gym.

This image immediately and clearly conveys the context of detention, and the idea that sport in the context of prison or youth detention changes the experience and pedagogy of sport. Lining up to leave when the activities are done, usually accompanied by pat-down searches from staff, is something Dan has observed at all the youth facilities he has visited (in both the US and Sweden, girls' and boys' institutions). Although it is not necessarily a universal part of sport in prisons or youth detention, it is certainly a common experience that is unique to the carceral setting. On the outside, when sport activities end, you might see people hanging around, chatting, perhaps a few are still playing or training. The participants might even go out for a pizza or some beers. But in carceral settings, sport ends abruptly, and no matter how fun or enjoyable the activity is, the return to institutional life is inescapable. In their narrative representation of sport in prison, [(45), p. 372] conclude that when sport is over, “Life in prison continues, inexorably.”

Thus, the image seeks to convey an important metaphor in the research: how sport activities are used to “ferry the time” (*att slussa tiden*) (32). Ferrying the time can be likened to an institutional pedagogy where the only clear aim with sport is to “busy the youth” to keep them (temporarily) calm or manageable. In this image we see this pedagogy as an aspect of everyday life in youth detention: where bodies are funneled or siphoned through series of locked doors – from one unit to another unit, from one activity to another. When any sport activity concludes, a similar routine ensues where the boys are lined up and then (typically) subjected to pat-down searches by staff. This generally happens after all activities in detention when youth are made to move from one location to another, for example at the end of class or at the end of a meal in the dining hall. They must wait for the metaphorical canal lock (sluss) to fill, as they go through the security routines, including being patted down under the suspicion of stealing something or concealing a possible weapon. The details of the illustration (i.e., clock) also communicate how time is experienced in confinement, going from one activity to the next, always with an underlying concern that the young people are calm or controlled [see (6)]. The illustrator added the clock on her own accord to emphasize these themes.

Like Forde (13), who realized the importance of time when drawing during ethnographic fieldwork, this theme appeared in Dan fieldwork too, albeit not exactly in the same way. Although the thesis was about sports, fieldwork life in detention was remarkably devoid of doing sports. Moreover, sports were often positioned as an escape from boredom and confinement in the units [see also (6, 46)]. Several students, for example, described sport and physical activity to break the monotony and at least “sit off” or “make use of the time.” In this way, sport activities were positioned as contrasting activities to life in a total institution generally. It was therefore important that not all the images in the thesis were depicting sport, but also the material conditions. When considering how to construct the illustrations, Dan wanted to convey the psych ward feel of some of the units at the homes. He was inspired by sensory ethnography (10), and wanted to capture the sterile feel, the particular smell, the low ceilings, the fluorescent light (and lack of natural light), and the sparse, particular kind of furniture, as described in the writing:

*After the ride we unlock and re-lock a series of doors leading to the common room in Sayyid's unit. The familiar smells of youth detention; recently mopped floors over a faint smell of sweat and cigarette smoke. The sun is blocked out and the room has a bluish, bright fluorescence. Two boys sit on the couch, watching daytime television: Cops (it's always Cops!). Our intrusion to an all-too-familiar boredom is welcomed*.

In this text, an experience of riding mountain bikes on a beautiful summer day is juxtaposed with the “thick” boredom of confinement at the units. However, life in the units was not always boring. On several occasions hanging with the boys and staff was rather fun and joyous. Choosing to create a warm image of boys and staff playing cards and having coffee, telling stories and laughing would have been possible, but that would misrepresent the youth's relentless experiences of being confined and looking for ways to “sit off” the time.

## Discussion—Reflections on illustrating an ethnography

In this paper, we have described and discussed the approach Dan used to illustrating an ethnography on sport in youth detention. In this discussion, we offer some reflections for illustrating ethnography, and some considerations for a methodology for doing so. Illustrations can have many possible purposes and benefits when supplementing ethnographic texts. One of the key considerations in this work had to do with *ethical* purposes, by which using illustrations instead of photos protected the confidentiality of people and places involved in the study, but also helped to humanize the youth participants. Additionally, illustrations might capture certain themes in a way that the details or realism of photos might distract from, and therefore have the analytical benefit of being able to focus on specific themes. Furthermore, another aim was to *affectively* convey the key findings or themes in the research. In particular, this meant to stimulate pedagogical reflection – i.e., that the readers reflect on the ways by which sport is – or might be – arranged and delivered in youth detention. According to van Manen (29), this can be considered the ultimate goal of pedagogical texts.

Indeed, researchers using illustrations and reflecting on why and how tend to adamantly argue for the benefits. For example, Herbst [(24), p. 84] concludes that graphics “are a tool, a final product and a method.” Like Forde (13) and Febres-Cordero et al. (16) she acknowledges the value of illustrating in that is stimulates reflection and analysis in several phases of the research. Herbst states that illustrations have implications for the discipline of anthropology more broadly: “It makes it accessible. It protects it. It opens up rather than closes it. It opens up new ways of thinking to ourselves, the ethnographers” [(24), p. 84]. Although we unquestionably agree, as our manuscript mirrors, we simultaneously reflect on the potential risks of using illustrations not least in terms of credibility. Because who should validate our research illustrations? Is it the research participants, the stakeholders, fellow academic peers, the artists, or is it some other category? Yes, images are powerful and useful means of communication, but they should also be recognized as part of visual genres of various sorts with innate connotations affecting what is communicated [see e.g., (9)]. We encourage future research to further address these issues.

Redirecting our attention from the risks to the benefits again, we think that illustrating texts can help make studies “more ethnographic” (2). Visual methods lend credibility to ethnographic work: images show that you have really been there, and that you understand what's going on. Plus, let us not forget that in a genre that is so textual images can be visually pleasing to the reader [c.f. (13, 24)]. Others who research sport in or as total institutions [c.f. (35)] might consider illustrations as a method for opening the door and inviting unfamiliar readers into contexts or subcultures that are closed or otherwise difficult to access [c.f. (16, 24)]. Norman (35) argues for a bricolage approach to researching total institutions – where researchers piece together various sources of information to gain insights to experiences within total institutions. Similarly, this paper shows that illustrations can be a useful method for quilting together thick description of sport in a total institution, in other words a *descriptive* purpose.

Ultimately, incorporating a thought-through visual method at the end of the text production was an important and successful contribution to the work. The illustrations were well-received by interested readers and stakeholders. However, the methodology was not without limitations or flaws, and could certainly be improved upon. There are some other important things missing from the illustrations. Although Dan was a participatory observer, he is absent from the illustrations. Images of practitioners working with the youth are also absent.

After the publication, he has also pondered on taking more risks, or undertaking other purposes in the illustrations?

To add to van Maanen's (23) different kinds of tales in ethnographic writing (realist, impressionist, and confessional), we think there are fruitful pathways in telling activist tales through illustrations. We think having artwork by the youth themselves would have not only added authenticity and legitimacy but could have strengthened a social justice purpose with the research. Incorporating the artwork of the imprisoned is not only important for visualizing critical perspectives on the power structures of total institutions, but it can also address the power (im)balances between researchers and participants in prison ethnography [c.f. (47)]. Likewise, visual methods in research with young people in vulnerable situations involving for example photo-voice or drawings [c.f. (48)] are being used to balance such power dynamics between participants and researchers. With more consideration, time, and resources, incorporating a youth-participatory wrinkle into the data collection and general ethnographic approach could have been interesting [c.f. (49)]. But, reflecting on the limitations of a novice researcher, extra wrinkles can muddle data collection and analysis. In what ways would a deliberate action research approach to the illustrations have impacted the main results of the research? It might have been tangential and therefore the illustrations, in hindsight, were adequately saved as final touch to the project.

As a concluding reflection, we hope this paper can help inspire others to employ illustration when representing their ethnographic tales. As with other mediums of qualitative analysis and writing, illustrations must be deliberately crafted, with thorough argumentative, theoretical, methodological, and creative considerations. Moreover, it could be a stimulating gateway into other visual-creative-critical methodologies for future research and in pedagogical life.

## Data availability statement

The raw data supporting the conclusions of this article will be made available by the authors, without undue reservation.

## Ethics statement

The studies involving human participants were reviewed and approved by Stockholm regional ethical review board. Written informed consent from the participants' legal guardian/next of kin was not required to participate in this study in accordance with the national legislation and the institutional requirements.

## Author contributions

All authors listed have made a substantial, direct, and intellectual contribution to the work and approved it for publication.

## Conflict of interest

The authors declare that the research was conducted in the absence of any commercial or financial relationships that could be construed as a potential conflict of interest.

## Publisher's note

All claims expressed in this article are solely those of the authors and do not necessarily represent those of their affiliated organizations, or those of the publisher, the editors and the reviewers. Any product that may be evaluated in this article, or claim that may be made by its manufacturer, is not guaranteed or endorsed by the publisher.

## Footnotes

^1^Captions have been herein provided in order to comply with journal requirements.
